# Dissection of *HY5/HYH* expression in *Arabidopsis* reveals a root-autonomous HY5-mediated photomorphogenic pathway

**DOI:** 10.1371/journal.pone.0180449

**Published:** 2017-07-06

**Authors:** Yonghong Zhang, Chen Li, Jingxuan Zhang, Jiajing Wang, Jingwei Yang, Yanxia Lv, Nian Yang, Jianping Liu, Xuanbin Wang, Gergo Palfalvi, Guodong Wang, Lanlan Zheng

**Affiliations:** 1 Laboratory of Medicinal Plant, School of Basic Medicine, Hubei University of Medicine, Shiyan, China; 2 Laboratory of Chinese Herbal Pharmacology, Oncology Center, Renmin Hospital, Hubei Key Laboratory of Wudang Local Chinese Medicine Research, Hubei University of Medicine, Shiyan, China; 3 Key Laboratory of Ministry of Education for Medicinal Plant Resource and Natural Pharmaceutical Chemistry, National Engineering Laboratory for Resource Developing of Endangered Chinese Crude Drugs in Northwest of China, College of Life Sciences, Shaanxi Normal University, Xi'an, China; 4 Tobacco Research Institute, Chinese Academy of Agricultural Sciences, Qingdao, Shandong, China; 5 Institute of Biology, Faculty of Sciences, University of Pécs, Pécs, Hungary; National Taiwan University, TAIWAN

## Abstract

ELONGATED HYPOCOTYL 5 (HY5), a member of the bZIP gene family, is a positive regulator of the light signaling pathway in *Arabidopsis thaliana*. Whereas the *hy5* mutant exhibits an elongated hypocotyl when grown in the light, the *hy5 homolog* (*hyh*) mutant does not. Although the functions of HY5 and HYH in light-mediated seedling development have been revealed, the tissue-specific expression patterns of *HY5* and *HYH* and their interconnected regulation are largely unknown. Here, we report that HY5 regulates *HYH* expression in roots and contributes to root growth under different light conditions. We generated *HY5* and *HYH* transcriptional and translational fusion reporter lines to investigate their expression patterns. *HY5* was constitutively expressed in all root tissues, while *HYH* was predominantly expressed in root xylem cells. Root growth after a dark-to-light transition was perturbed in the *hy5* and *hy5hyh* mutant lines, but not in the *hyh* mutant line, indicating that HY5 plays a major role in light-regulated root growth. Light-induced *HY5*/*HYH* expression occurred autonomously in roots. *HYH* expression in roots was decreased in the *hy5* mutant, suggesting that HY5 regulates *HYH* expression. Collectively, these results indicate that an organ-specific HY5-mediated pathway controls root photomorphogenic development independently of light signaling in the shoot.

## Introduction

Light is essential for plant growth and development, providing energy for photosynthesis and regulating seedling photomorphogenesis, seed germination, shade avoidance, and photoperiod responses [1,2]. Among these developmental processes, photomorphogenesis is one of the most extensively studied [3]. Molecular genetic studies have revealed various regulators downstream of photoreceptors [4,5], including the RING E3 ubiquitin ligase CONSTITUTIVE PHOTOMORPHOGENIC 1 (COP1), a major integrator of light responses [6]. COP1 ubiquitinates two downstream basic domain/leucine zipper (bZIP) transcription factors, ELONGATED HYPOCOTYL 5 (HY5) and HY5 HOMOLOG (HYH), and mediates their degradation [7–9]. HY5 was the first transcription factor shown to be involved in promoting photomorphogenesis, and has been extensively studied [10–12]. Although mutations in *HY5* result in elongated hypocotyls even under constant light, the *hyh* mutant exhibits no obvious phenotype [7,13,14]. Both of these genes are required for UV RESISTANCE LOCUS8 (UVR8)-regulated UV-B signaling [15]. PROLIFERATING CELL FACTOR 2 (TCP2) was reported to transcriptionally activate *HY5* and *HYH* expression via the cryptochrome 1 (CRY1)-mediated photosensory signaling pathway [16].

In nature, the root systems of most terrestrial plants are underground and in darkness; however, roots contain photoreceptors that may detect ambient light through the soil [17,18], and can also respond to direct illumination when grown in transparent Petri dishes [19–21]. The root cap is reported to be essential for the photophobic root behavior in maize, which implies that a local light response exists in roots [22]. The SCAR/WAVE complex, which facilitates nucleation of new short F-actin branches from existing filaments, mediates light-induced root elongation independently from the shoot in Arabidopsis [23]. In contrast to the well-studied light responses of aboveground tissues, the mechanism by which light regulates root development is not well understood. Previous work has suggested that COP1-mediated auxin transport is involved in adapting root growth to the ambient light conditions [24], while the light-dependent signaling of HY5, the best-characterized target of COP1-mediated proteolysis, plays an important role in PIN2 intracellular distribution in roots [25]. Recently, it was reported that aboveground light is directly transmitted through the stem and activates phytochrome B in the roots, triggering HY5-mediated light responses [26]. Moreover, HY5 mediates the promotion of root growth and nitrate uptake in response to light, acting as a shoot-to-root mobile signal [27]. However, the mechanism by which HY5 regulates root photomorphogenesis is largely unknown. Root growth in response to light is therefore mostly influenced by shoot-derived signals; however, we speculate that a root-autonomous light response also exists.

There is growing evidence to suggest that *HY5* and *HYH* expression is upregulated by light in the seedling [7,9,28]. However, their expression in roots has yet to be fully elucidated. A genome-wide study using chromatin immunoprecipitation with DNA chip hybridization (ChIP-chip) revealed that the *HYH* promoter has putative binding sites for HY5 [10]. A number of HY5 putative target genes identified in the ChIP-chip study were confirmed using a yeast one-hybrid (Y1H) assay and/or chromatin immunoprecipitation, including *COP1* [29], *FAR-RED ELONGATED HYPOCOTYL1* (*FHY1*) and its homolog *FHY1-LIKE* (*FHL*) [12], and the nitrate uptake gene *NITRATE TRANSPORTER 1*.*1* (*NRT1*.*1/CHL1*) [30]. Elucidating the regulatory relationship between *HY5* and *HYH* is a key step in deciphering the response of roots to light.

Here, we investigated the expression patterns of *HY5* and *HYH*, and found that HY5 directly regulates *HYH* expression in roots. We confirmed that *HY5*, and to a lesser extent *HYH*, contributes to root growth under dark/light conditions.

## Materials and methods

### Plant materials and growth conditions

All *Arabidopsis* mutants used in this study were in the Columbia (Col-0) background. The *hy5-2 (*SALK_056405), *hy5-51* (SALK_096651), and *hyh* (WISCDSLOX253D10) mutants were obtained from the Nottingham Arabidopsis Stock Centre. The primers used for genotyping are listed in S1 Table. Seedlings were germinated on half-strength Murashige and Skoog (MS) agar plates and then incubated in a near-vertical position at 22°C under continuous white light (120 μmol m^−2^ s^−1^) or darkness (packed with foil) in a plant growth chamber for 7 d or as indicated.

### Plasmid construction and plant transformation

To generate *HY5* and *HYH* promoter fusion constructs, promoter regions upstream of the start codons of *HY5* and *HYH* were fused to a *GUS* (*β-Glucuronidase*) or *ERGFP* (Endoplasmic Reticulum-Green Fluorescent Protein) reporter gene and nopaline synthase terminator in the pGreenII-0229 plasmid (www.pgreen.ac.uk). To create protein fusion constructs of *HY5*::*HY5*:*GFP* and *HYH*::*HYH*:*GFP*, genomic fragments of *HY5* and *HYH*, including the respective 700-bp and 3000-bp promoter region (up to the next upstream gene), were fused in frame to the N terminus of the *GFP* reporter gene engineered in pGreenII-0229. The resulting constructs were then transformed into WT and/or *hy5* plants using the floral dip method [31]. The primers used for cloning are listed in S1 Table.

### Phenotypic and expression analyses

GUS staining was performed as previously described [24]. Briefly, samples of 7-day-old seedlings were incubated in assay buffer [50 mM sodium phosphate (pH 7.0), 10 mM EDTA, 1 mM potassium ferricyanide, 1 mM potassium ferrocyanide, 0.1% Triton X-100, 20% methanol, and 0.5 mg/mL X-Gluc A] at 37°C until sufficient staining was observed. GUS activity was analyzed with a Nikon 80i microscope using Nomarski interference contrast optics. To prepare cross sections, roots were embedded in 2% agarose, fixed in 4% paraformaldehyde, dehydrated in an ethanol series, embedded in Technovit 7100 (Heraeus Kulzer, EBSciences), and sectioned at 15 μm. For analyses of hypocotyl and root phenotypes, 4-day-old etiolated seedlings were transferred to light for additional days (one additional day for hypocotyl and eight additional days for root). Root tip positions were marked with a pen each day. Seedlings were imaged with a Canon 40D camera and root length was measured using ImageJ (http://rsbweb.nih.gov/ij/). Quantitative analyses of the root apical meristem (RAM) were performed as previously described [24]. Statistical analyses (pairwise two-tailed Student’s *t-*test) were performed using Microsoft Excel. Confocal imaging was performed using a Leica TCS SP2 microscope. Propidium iodide (Sigma-Aldrich; 10 μg/mL dissolved in water) was used to counterstain root cells. Epifluorescence images were taken using a Leica M205FA stereomicroscope. The roots of the seedlings were submerged in water on the microscope slide before the coverslip was placed over the root. For all light/dark transition experiments, seedlings were grown in continuous light or darkness for 5 d. Seedlings were then transferred to the opposite condition for 24 h. Samples were then imaged under a confocal microscope or stereomicroscope. For the decapitation experiment, 5-day-old light- and dark-grown seedlings were decapitated by a double edge razor blade before the light/dark transition. Each experiment was repeated at least three times.

### Quantitative Reverse Transcription Polymerase Chain Reaction (qRT-PCR)

RNA was isolated using Tranzol (Transgene) in accordance with the manufacturer’s protocol. cDNA was prepared using a PrimeScript RT Reagent Kit (RR047A, Takara). Relative expression levels were determined by qRT-PCR using the ABI 7500 Real-Time PCR System or the ViiA™ 7 Real-Time PCR System (Life Technologies). *EF1α* was used as reference gene for normalization. The primer sequences used for qRT-PCR analysis are listed in S1 Table.

### Yeast one-hybrid assays (Y1H)

For yeast one-hybrid assays, plasmids harboring AD (activation domain) fusions were cotransformed with the LacZ reporter gene driven by four fragments (frag 1–frag 4) of the *HYH* promoter into the yeast strain EGY48 [12,32]. Transformants were grown on proper dropout plates containing X-gal (5-bromo-4-chloro-3- indolyl-β-D-galactopyranoside) for blue color development. Yeast transformation was conducted as described in the Yeast Protocols Handbook (Clontech). The primers used for cloning are listed in S1 Table.

### Electrophoretic mobility shift assay (EMSA)

The full-length coding region of *HY5* was first cloned in frame into the SalI–EcoRI sites of the pSPUTK *in vitro* transition vector (Stratagene). The HY5 protein was synthesized using a TNT SP6 Quick Coupled Transcription/Translation System (Promega) according to the manufacturer’s instructions in a total volume of 10 μL [33]. 3’ FAM oligonucleotides were synthesized and labeled by the Shanghai Sangon Company (S1 Table). To generate double- stranded oligos, an equal amount of the complementary single-stranded oligos was mixed, heated to 95°C for 2 min, and annealed by gradually cooling down to 25°C. To perform the EMSA, HY5 was pre-incubated with the Binding buffer [1 μg poly(dI-dC), 10 mM Tris-HCl (pH 7.5), 50 mM KCl, 1 mM DTT, 2.5% glycerol, and 5 mM MgCl_2_] at room temperature for 20 min. For the competition assay, non-labeled probe was incubated with protein and Binding buffer at room temperature for 20 min. Then, 1 μL of 3’ FAM-labeled probe (10 μM) was added and samples were incubated at room temperature for 20 min. The samples were then subjected to electrophoresis on 6% PAGE gels running with 0.5× Tris-borate- EDTA buffer at 4°C in darkness for 1 h. The fluorescence signaling was captured using a FLA-5100 fluorescent image analyzer (Fuji).

### Accession numbers

Sequence data from this article can be found in the GenBank/EMBL libraries under the following accession numbers: *HY5*, AT5G11260; *HYH*, AT3G17609.

## Results

### Expression pattern of *HY5* and *HYH* in response to light

**Fig 1:**
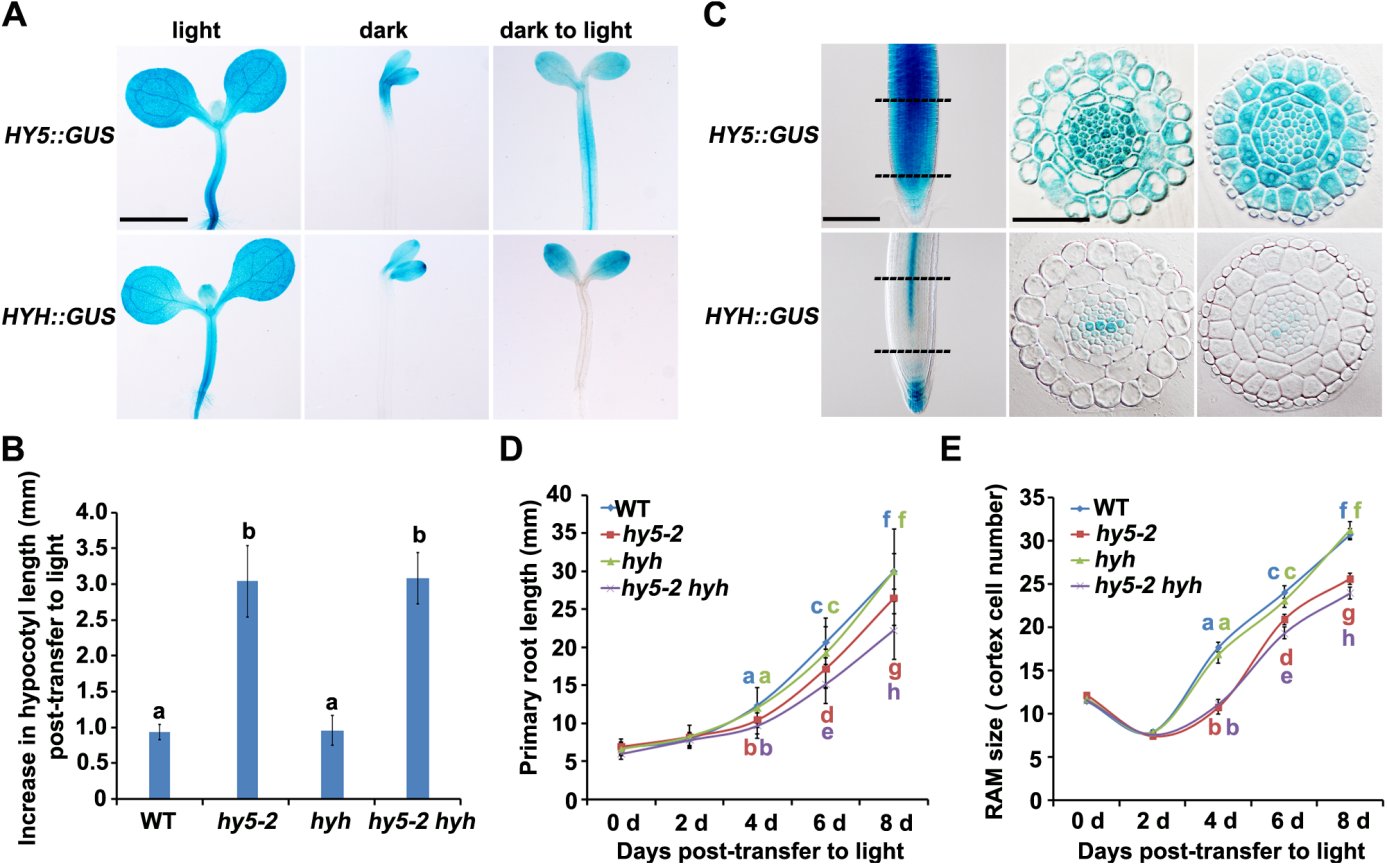
Expression patterns of *HY5* and *HYH* and phenotypic analysis in response to light. (**A**) Expression patterns of *HY5*::*GUS* and *HYH*::*GUS* reporter lines in the shoots of plants grown under light, dark, and dark-to-light conditions for 7 d. Scale bar: 1 cm. (**B**) The increase in hypocotyl length of four-day-old etiolated wild-type (WT), *hy5-2*, *hyh*, and *hy5-2 hyh* seedlings exposed to light for an additional day (n>12). Error bars represent SD (standard deviation). (**C**) Expression patterns of *HY5*::*GUS* and *HYH*::*GUS* reporter lines in the roots of plants grown in the light for 7 d. The middle and right panels are cross sections of the roots shown in the left panels, at positions denoted by the upper and lower dashed lines, respectively. Scale bar: 50 μm. (**D and E**) Primary root length (D) and root apical meristem (RAM) size (E) of four-day-old etiolated WT, *hy5-2*, *hyh*, and *hy5-2 hyh* seedlings transferred to constant light (n = 20). Error bars represent SD. In C, D, and E, error bars with different letters indicate a significant difference at p<0.05 (*t* test).

To investigate the expression patterns of the light signaling master regulator *HY5* and its homolog *HYH*, we constructed their promoter reporter lines, *HY5*::*GUS* and *HYH*::*GUS*, respectively. These lines revealed that the two genes exhibited a similar expression pattern in shoots grown in the light (Fig 1A and S1 Fig). In dark-grown etiolated seedlings, both lines showed GUS activity in the cotyledons; however, *HY5* was expressed in the upper hypocotyl, whereas *HYH* was not (Fig 1A). Immunoblot analysis of total protein extracts from intact seedlings previously revealed that *HY5* and *HYH* were induced after light treatment [7,9]. We confirmed this finding using the *HY5*::*GUS* line, in which *HY5* was expressed in the entire hypocotyl following a dark-to-light transition. By contrast, *HYH* was not induced in the hypocotyl (Fig 1A). The increase in hypocotyl elongation of etiolated seedlings after light treatment was enhanced in the *hy5-2* and *hy5-2 hyh* mutant lines, but not in the *hyh* single mutant (Fig 1B and S1 Data). These results confirm the previous findings that HY5, but not HYH, is a major repressor of hypocotyl elongation during shoot photomorphogenesis.

*HY5* and *HYH* were shown to be differentially expressed in the roots (Fig 1C). In plants grown in the light, *HY5* was constitutively expressed in all root tissues, while *HYH* was predominantly expressed in the root vascular tissues and columella cells (Fig 1C). A root cross section of the *HYH*::*GUS* line showed GUS activity in the xylem cells (Fig 1C). The expression of *HY5* and *HYH* in roots suggests that the HY5-mediated light signaling pathway may also function in root development. We measured the root length of wild-type (WT), *hy5-2*, *hyh*, and *hy5-2 hyh* lines under dark conditions. For the nine days measured, there were no significant differences in the *hy5-2*, *hyh*, and *hy5-2 hyh* lines compared with the WT in darkness (Figure A in S2 Fig and S2 Data). These observations indicate that HY5 and HYH do not function in early root development under dark conditions. Given that *HY5* and *HYH* were induced by light, we also examined the root length and cortex cell number of WT, *hy5-2*, *hyh*, and *hy5-2 hyh* plants following a dark-to-light transition. After transferring four-day-old etiolated seedlings to the light, the *hy5-2* and *hy5-2 hyh* lines, but not the *hyh* mutant, exhibited shorter roots and smaller root apical meristems than the WT (Fig 1D and 1E and S1 Data). These results indicate that *HY5*, but not *HYH*, promotes root elongation after a transition from dark to light conditions. In addition, the *hy5-2 hyh* double mutant showed a more severe root phenotype than *hy5-2* after eight days of growth in light (Fig 1D and 1E and S1 Data). When grown in the light for 9 to 10 d, the *hy5-2* and *hy5-2 hyh* mutants had shorter roots than the WT and *hyh* lines, with the shortest roots observed in the double mutant (Figure B in S2 Fig and S2 Data). These results indicate that *HYH* plays a role in late root development in response to light. The observation that *HY5* has different functions in shoot and root elongation suggest that these systems respond differently to light. Although these homologs exhibit different expression patterns, the root phenotypes suggest that there is functional redundancy between them.

### Light induces *HY5* and *HYH* transcripts and proteins in roots

**Fig 2:**
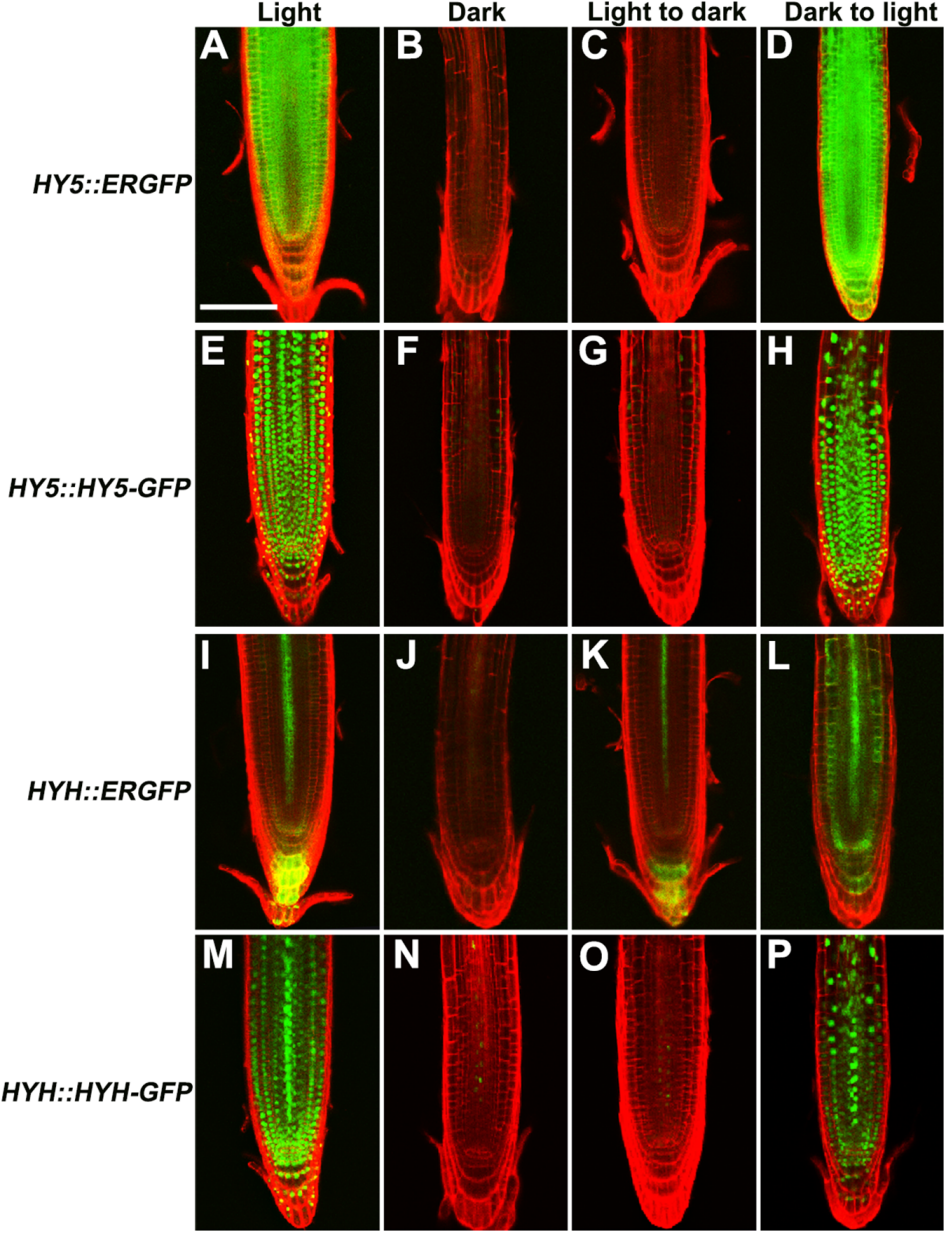
*HY5* and *HYH* transcripts and proteins were induced in the root in response to light. (**A–H**) Expression of *HY5* promoter fusion (**A–D**) and protein fusion (**E–H**) lines in roots under light, dark, light-to-dark and dark-to-light transition conditions. (**I–P**) Expression of the *HYH* promoter fusion (**I–L**) and protein fusion (**M–P**) lines in roots under light, dark, light-to-dark, and dark-to-light transition conditions. Scale bar represents 50 μm.

Under the typical agar-plate culture system, the entire *Arabidopsis* seedling, including the root, is exposed to light; however, the light response and corresponding gene expression patterns of roots have not been extensively examined in previous photomorphogenic studies [5,19–21]. In the present study, we generated the *HY5* and *HYH* transcriptional/translational GFP reporter lines (*HY5*::*ERGFP* and *HY5*::*HY5-GFP*, *HYH*::*ERGFP*, and *HYH*::*HYH-GFP*) to investigate their expression in roots in response to light. In addition, the GFP fusions driven by the cauliflower mosaic virus 35S promoter were generated and subsequently introduced into WT or *hy5-2* mutant backgrounds and confirmed to function properly using a hypocotyl elongation assay (S3 Fig and S3 Data). Both *HY5* and *HYH* transcripts and proteins accumulated in roots under the light and dark-to-light transition conditions, but were suppressed in darkness and under the light-to-dark transition conditions (Fig 2). A qRT-PCR analysis determined that the transcriptional levels of *HY5* and *HYH* were 4.9 ± 0.5 (mean ± SD) and 4.2 ± 0.3 times greater in the light than in darkness, respectively (S4 Fig and S4 Data). These results indicate that both *HY5* and *HYH* levels are altered in response to light in the root.

### Root-autonomous regulation of *HY5* and *HYH* expression in response to light

**Fig 3:**
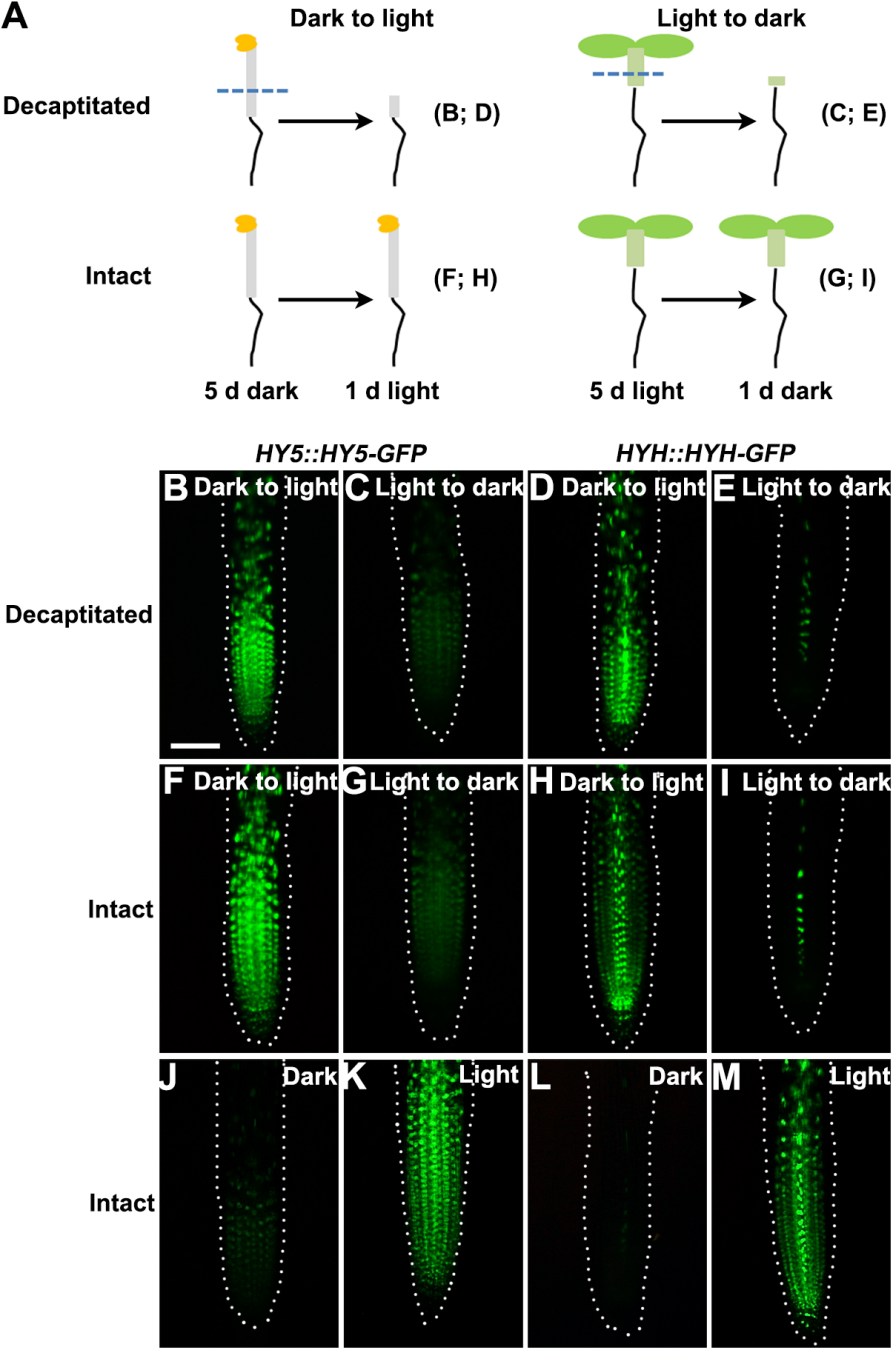
Root-autonomous regulation of *HY5* and *HYH* expression in response to light. (**A**) Diagram of the decapitation procedure. Dashed lines mark the cutting positions. Letters in brackets represent the corresponding panels. (**B**,**C**,**F**,**G**) Expression of HY5 protein fusion lines in roots of decapitated or intact plants under dark-to-light and light-to-dark transitions. (**D**,**E**,**H**,**I**) Expression of HYH protein fusion lines in roots of decapitated or intact plants under dark-to-light and light-to-dark transitions. (**J**,**K**) Expression of HY5 protein fusion lines in the roots of intact seedlings under dark and light growth conditions. (**L**,**M**) Expression of HYH protein fusion lines in the roots of intact seedlings under dark and light growth conditions. Images were acquired using a fluorescence stereomicroscope. The dotted line indicates the root outline. Scale bar in (B): 50 μm.

Changes in *HY5* and *HYH* expression in roots following exposure to light might be regulated by a signal from the shoot or, alternatively, might be root autonomous. To test these hypotheses, we examined the expression patterns of *HY5* and *HYH* in roots after removing the shoots. In decapitated *HY5*::*HY5-GFP* and *HYH*::*HYH-GFP* lines, the expression levels of both genes increased and decreased when plants were subjected to the dark-to-light and light-to-dark transition conditions, respectively (Fig 3). These responses were similar in both decapitated plants and intact seedlings, indicating that *HY5* and *HYH* expression in the roots respond to light in a root-autonomous manner.

### *HYH* expression in roots is regulated by HY5

**Fig 4:**
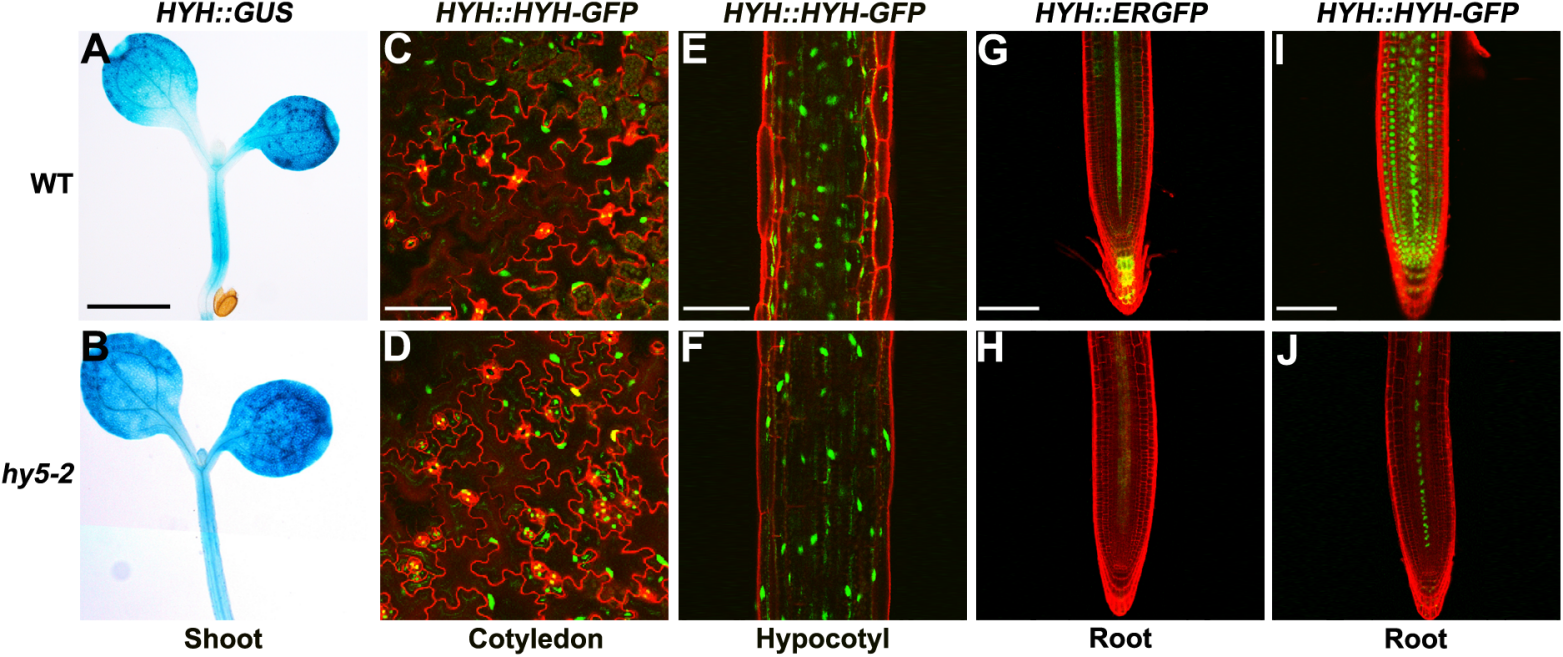
Expression of *HYH* transcripts and proteins in the shoots and roots of WT and *hy5-2* seedlings. (**A**,**B**) Expression of the *HYH*::*GUS* fusion in shoots of 7-d-old light-grown WT (A) and *hy5-2* (B) seedlings. Scale bar in (A,B): 1 mm. (**C**,**D**) Expression of the *HYH*::*HYH-GFP* fusion in the cotyledons of WT (C) and *hy5-2* (D) seedlings. (**E**,**F**) Expression of the *HYH*::*HYH-GFP* fusion in the hypocotyls of WT (E) and *hy5-2* (F) seedlings. (**G**,**H**) Expression of the *HYH*::*ERGFP* fusion in the roots of WT (G) and *hy5-2* (H) seedlings. (**I**,**J**) Expression of the *HYH*::*HYH-GFP* fusion in the roots of WT (I) and *hy5-2* (J) seedlings. Scale bars in (C–J): 50 μm.

To further study the functions of *HY5*/*HYH* in root development in response to light, we investigated the interconnected regulation of *HY5* and *HYH*. Given that *HY5* and *HYH* expression were repressed under dark conditions, we first examined the expression of these genes in the reciprocal mutants using qRT-PCR and reporter lines grown in the light. *HY5* transcript levels were similar in both the shoots and roots of the *hyh* mutant compared with those observed in the WT (Figure A in S5 Fig and S5 Data). Examination of the *HY5*::*HY5-GFP* line indicated that there was no difference in the level of HY5 protein in the roots of the WT and the *hyh* mutant (Figure B in S5 Fig), suggesting that *HY5* is not regulated by *HYH*. In a previous study, *hy5-2* was demonstrated to be a knockout mutant [34]. However, to exclude any possible T-DNA multi-insertion effects in this line, the experiments were repeated using another T-DNA insertion line, *hy5-51* (SALK_096651), which has also been repeatedly used in published research [35]. The *hy5-51* mutant is also a knockout line, and exhibits the same phenotype as *hy5-2* (Figure A to C in S6 Fig). The *HYH* transcript level was repressed in the root but not the shoot of both *hy5* mutants (Figure D and E in S6 Fig and S6 Data). To confirm that *HYH* is repressed in the *hy5-2* mutant, we examined the *HYH* promoter reporter and protein reporter lines in the *hy5-2* background and compared these with the WT. The promoter activity of *HYH*, represented by *HYH*::*GUS*, exhibited no differences in the shoots of the WT and the *hy5-2* mutant (Fig 4A and 4B). Similarly, the levels of HYH protein in the cotyledons and hypocotyls were unchanged in the WT and *hy5-2* mutant seedlings (Fig 4C to 4F). By contrast, *HYH* transcript and protein levels in the roots were decreased in the *hy5-2* mutant compared with the WT (Fig 4G to 4J). These results indicate the existence of a root-specific HY5-mediated pathway that regulates *HYH* expression.

**Fig 5:**
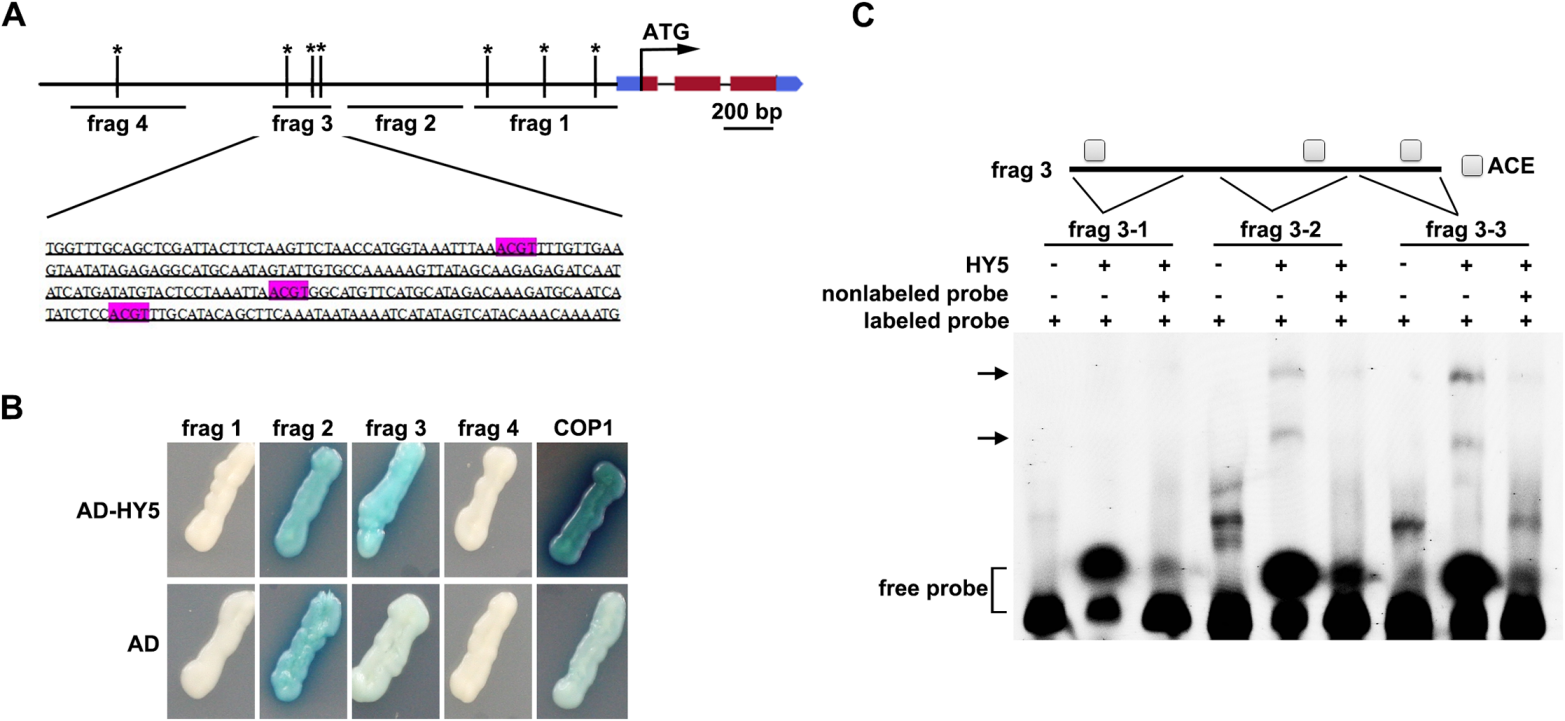
HY5 binds to the *HYH* promoter. (**A**) Schematic model of *HYH* genomic sequences. Four fragments of the *HYH* promoter region (frag 1–4; indicated by lines) were selected for use in a yeast one-hybrid assay (Y1H). Asterisks represent the putative HY5 binding sites. The “ACGT” sequences targeted by HY5 are highlighted for frag 3. (**B**) Y1H demonstrating that HY5 binds to the *HYH* promoter at frag 3. *COP1* serves as a positive control [27] and the empty vector expressing the AD domain alone is the negative control. (**C**) An EMSA confirmed the binding of HY5 to the promoter of *HYH*. Scheme showing the structure of the frag 3 region. The squares indicate ACGT-containing elements (ACE) in frag 3. The corresponding electrophoretic mobility shift assay (EMSA) probes are illustrated as frags 3–1, 3–2, and 3–3. HY5 was produced in an *in vitro* expression system. The labeled probes were 3' FAM oligonucleotides, while non-labeled probes served as competitors. Arrows indicate the shifted bands.

A previous genome-wide study showed that *HYH* is one of the targets of the HY5 transcription factor [10], suggesting that *HYH* is regulated by HY5. To confirm this, we performed a yeast one-hybrid (Y1H) assay. HY5 has been shown to bind to ACGT-containing elements (ACEs) and promote the activation of light-induced genes [10,12,36]. Several putative HY5 binding sites were selected from the *HYH* promoter region for the Y1H assay (Fig 5A). The expression of the *LacZ* reporter gene was determined by the production of a blue colored colony on the X-gal plates. We found that HY5 bound to fragment 3 (frag 3) of the *HYH* promoter region, which contains three ACEs (Fig 5B). Although frag 1 and frag 4 also contain putative HY5 binding sites, they exhibited no binding activity in our Y1H assay. However, frag 2 showed strong trans-activation activity in yeast cells, because the negative control also appeared blue. To further confirm the binding activity at frag 3, three probes containing one single ACE each were designed and used to perform an electrophoretic mobility shift assay (EMSA; Fig 5C). The EMSA results indicated that HY5 could bind to the frag 3–2 and frag 3–3 regions of fragment 3. Collectively, our results indicate that HY5 binds directly to the *HYH* promoter at the frag 3 region.

## Discussion

The roles of HY5 in plant photomorphogenesis have been extensively studied, with a major focus on the shoot system [10,14,37]. Furthermore, large-scale omics data support the notion that HY5 is a key signal integration point during dark-to-light transitions [10,36,38]. It was recently demonstrated that phyB-activated HY5 mediates cellular responses to light in the root, and is important for primary root growth and root gravitropism [26,39]. HY5 has been reported to be a shoot-to-root mobile signal that mediates the light-responsive coupling of shoot growth and carbon assimilation with root growth and nitrogen uptake in *Arabidopsis* [27]. These findings suggest that *HY5* expression in the root affects both its growth and the expression of other genes in this tissue through two distinct pathways [26,27]. Nevertheless, our knowledge of the molecular mechanisms underlying root photomorphogenesis is fragmented. The accelerated root growth of *hy5* was reported to be associated with auxin signaling [13]; however, although HY5 is known to integrate the light and hormone signaling pathways [40], the details of this crosstalk are unclear.

In this study, *HY5* and *HYH* expression patterns were examined at the tissue level and under various light/dark growth conditions using GUS and GFP reporter lines (Fig 1A and 1B, Fig 2 and S1 Fig). Generally, both *HY5* and *HYH* showed similar expression patterns in the shoots in both darkness and in the light (Fig 1A and S1 Fig). Unlike *HY5*, *HYH* was not expressed in hypocotyls following the dark-to-light transition (Fig 1A), suggesting that *HY5*, but not *HYH*, contributes to hypocotyl elongation. Interestingly, *HY5* and *HYH* had distinct expression patterns in the root; *HY5* was expressed in all root tissues, while *HYH* was predominantly expressed in the root vascular tissues and the root cap (Fig 1C). A phenotypic analysis of root length in the WT and *hy5-2*, *hyh*, and *hy5-2 hyh* mutants indicated that growth of the primary root of the *hy5-2* mutant, but not the *hyh* mutant, was suppressed during the dark-to-light transition, with the most severe short-root phenotype observed in the *hy5-2 hyh* double mutant (Fig 1D and 1E and S2 Fig). These results indicate that *HY5* and *HYH* function in root development during de-etiolation.

Previous immunoblot analyses of total protein extracts from intact seedlings revealed that the expression of both *HY5* and *HYH* is induced by light [7,9]. In the present study, we used reporter lines to provide direct tissue-level evidence that the expression of both *HY5* and *HYH* in the roots is induced by light (Fig 2). Additionally, decapitation experiments suggested that the root response of *HY5* and *HYH* to light occurs in a root-autonomous manner (Fig 3). In combination with the root phenotype of the *hy5-2* and *hy5-2 hyh* mutants, our results indicate that root growth is regulated by the HY5/HYH-mediated light signaling pathway.

Roots may perceive light signals directly by photoreceptors [17,18]. Extensive studies showed that interactions between cryptochrome and phototropin play a key role in root phototropism and architecture [18,41–43]. Recent studies have shown that HY5 positively regulates the activity of its own promoter in a tissue-specific manner [44], and that HY5 and HYH bind to the T/G-box in the *HY5* promoter and act redundantly to induce *HY5* expression upon UV-B exposure [45]. An immunoblot analysis indicated that HYH protein levels are decreased in the *hy5* mutant and that HYH and HY5 might heterodimerize with each other *in vivo* [7]. To further elucidate the interconnected regulation of HY5 and HYH, we examined the reciprocal expression of *HY5* and *HYH* in their mutants using qRT-PCR and reporter lines grown in the light (Fig 4 and S3 Fig). Our findings indicated that *HYH* expression in roots is regulated by HY5. These results, in addition to previous immunoblot data [7], further demonstrate that the suppression of HYH in the *hy5* mutant is achieved in a root-specific manner. Similar to the regulation of *HYH*, the chalcone synthase gene (*CHS1*) was previously shown to be strongly induced by light in a HY5-dependent pathway, and the induction of *CHS1*::*GUS* was also specific to the root [8].

The results of Y1H and EMSA indicated that HY5 directly binds to a particular section (frag 3) of the *HYH* promoter that contains putative HY5 binding sites (ACEs) (Fig 5). Consistently, although *HY5* and *HYH* have additive effects in hypocotyl elongation [7], the *hy5* and *hy5hyh* mutants exhibited opposite lateral root architecture phenotypes [13]. Our findings suggest different pathways might be involved in the light-induced expression of *HYH* in the roots and shoots. We speculate that a possible unidentified factor might regulate HY5 and *HYH* during shoot photomorphogenesis. Further research is needed to fully elucidate these mechanisms.

Light is an important environmental factor influencing root growth. Long-distance signal transduction has long since been thought to be an important mechanism for the light response in roots [21], and auxin is the master signaling molecule in this pathway [24,25]. Furthermore, root apices generate massive amounts of reactive oxygen species (ROS) after only a few seconds of illumination [19,46]. Since roots grow continuously when placed in transparent Petri dishes, it was concluded that ROS, as a stress introduced by light, stimulates root growth [19]. How roots integrate shoot-derived and local signals is therefore fundamental for plant development. In previous photomorphogenic studies, the expression patterns of *HY5* and *HYH* were not adequately dissected, since both the roots and shoots were exposed to light. In the present study, we have revealed a tissue-specific HY5-mediated pathway that regulates light-induced root growth independently of light signaling in the shoot. Further work is needed to reveal the molecular components involved in this process.

## Supporting information

S1 Fig Expression patterns of *HY5* and *HYH* in two additional T2 transgenic promoter fusion and protein fusion lines. Seven-day-old light-grown (**A–D**) *HY5*::*GUS*; (**E–H**) *HYH*::*GUS*; (**I–L**) *HY5*::*ERGFP*; (**M–P**) *HYH*::*ERGFP*; (**Q–T**) *HY5*::*HYH5-GFP*; and (**U–X**) *HYH*::*HYH-GFP* seedlings. Numbers (#2, #3) on the upper right indicate individual T2 lines. Scale bars in A, C, E, G, I, K, M, and O: 1 cm. Scale bars in the remaining panels: 50 μm.
(TIF)

S2 Fig Primary root lengths of wild-type, *hy5-2*, *hyh*, and *hy5-2 hyh* lines grown in darkness and light. (**A**) Primary root length of the wild-type (WT), *hy5-2*, *hyh*, and *hy5-2 hyh* lines grown in darkness (n = 20). Error bars represent SD. (**B**) Primary root length of the WT, *hy5-2*, *hyh*, and *hy5-2 hyh* lines in the light (n>20). Error bars represent SD. Bars with different letters are significantly different at p<0.05 (*t* test).
(TIF)

S3 Fig Functional analysis of *HY5* and *HYH* translational *GFP* fusion lines. The hypocotyl length of 6-d-old light-grown wild type (WT), *hy5-2* mutant, and transgenic seedlings in the WT or *hy5-2* mutant background. Error bars represent SD (n>40). Bars with different letters are significantly different at p<0.05 (*t* test).
(TIF)

S4 Fig Expression level of *HY5* and *HYH* in roots under dark and light growth conditions. qRT-PCR quantification of *HY5* and *HYH* transcripts. Error bars represent SD from three independent experiments. *HY5* and *HYH* transcripts were normalized to the *EF1α* gene. ** P< 0.01, *t* test.
(TIF)

S5 Fig Expression level of *HY5* in the shoot and root of wild-type and *hyh* seedlings. (**A**) qRT-PCR quantification of *HY5* transcripts in the shoots and roots of wild-type and *hyh* plants. Error bars represent SD from three independent experiments. *HY5* transcripts were normalized to the *EF1α* gene. (**B**) Expression pattern of the HY5 protein fusion line in roots of wild-type and *hyh* plants. The dotted line indicates the root outline. Scale bar: 50 μm.
(TIF)

S6 Fig Expression level of *HYH* transcripts in the shoots and roots of WT and *hy5* mutants. (**A**) Schematic diagram showing the genomic structure of *HY5*. Gray boxes represent exons and horizontal lines represent introns. Arrowheads indicate the location of T-DNA insertion sites in the *HY5* gene. ATG, start codon; TGA, stop codon. (**B**) Reverse transcriptase polymerase chain reaction (RT-PCR) analysis of *HY5* expression in wild-type (WT) plants and the *hy5-51* mutant. *ACT2* serves as reference. (**C**) Lateral root phenotypes of 12-d-old light-grown WT, *hy5-2*, and *hy5-51* seedlings. Scale bar: 1 cm. (**D**,**E**) qRT-PCR quantification of *HYH* transcripts in the shoots and roots of the WT and *hy5* mutant. Error bars represent SD from three independent experiments. *HYH* transcripts were normalized to the *EF1α* gene. ** P< 0.01, *t* test.
(TIF)

S1 Table Primers used in the study. (DOCX)

S1 Data Mutant phenotypic analysis in response to light, related to Fig 1B, 1D and 1E. (XLSX)

S2 Data Mutant phenotypic analysis under dark conditions, related to S2 Fig. (XLSX)

S3 Data Functional analysis of *HY5* and *HYH* translational *GFP* fusion lines, related to S3 Fig. (XLSX)

S4 Data Expression level of *HY5* and *HYH* in roots under dark and light growth conditions, related to S4 Fig. (XLSX)

S5 Data Expression level of *HY5* in the shoot and root of wild-type and *hyh* seedlings, related to S5A Fig. (XLSX)

S6 Data Expression level of *HYH* transcripts in the shoots and roots of WT and *hy5* mutants, related to S6D and S6E Fig. (XLSX)
