# Supplementary figures and images for: Dissection of HY5/HYH expression in Arabidopsis reveals a root-autonomous HY5-mediated photomorphogenic pathway
Source: PLoS One. 2017 Jul 6;12(7):e0180449. doi: 10.1371/journal.pone.0180449 (PMC5500333; doi:10.1371/journal.pone.0180449)

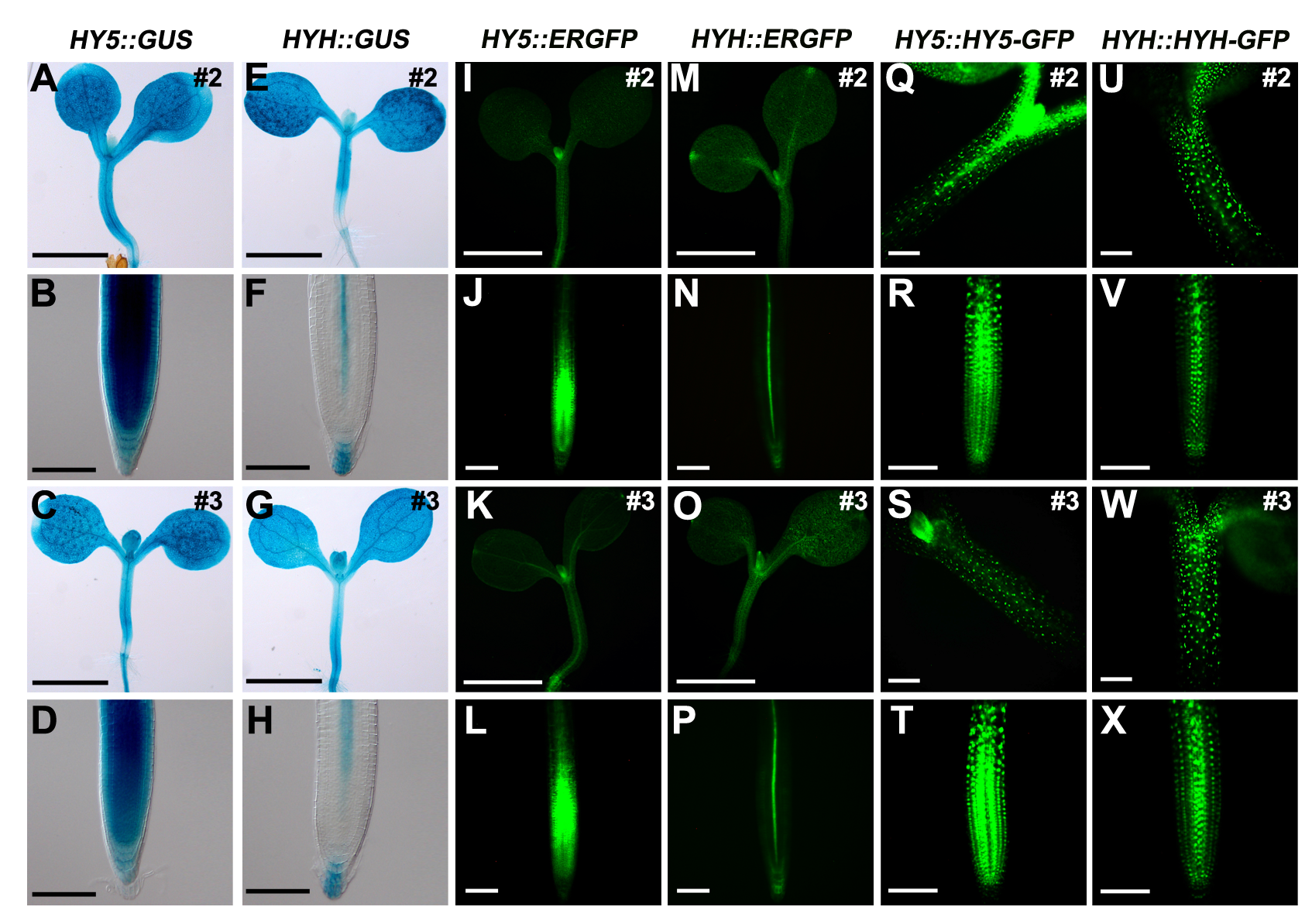

Supplement: S1 Fig — Seven-day-old light-grown (A–D) HY5::GUS; (E–H) HYH::GUS; (I–L) HY5::ERGFP; (M–P) HYH::ERGFP; (Q–T) HY5::HYH5-GFP; and (U–X) HYH::HYH-GFP seedlings. Numbers (#2, #3) on the upper right indicate individual T2 lines. Scale bars in A, C, E, G, I, K, M, and O: 1 cm. Scale bars in the remaining panels: 50 μm. (TIF) [file pone.0180449.s001.tif]

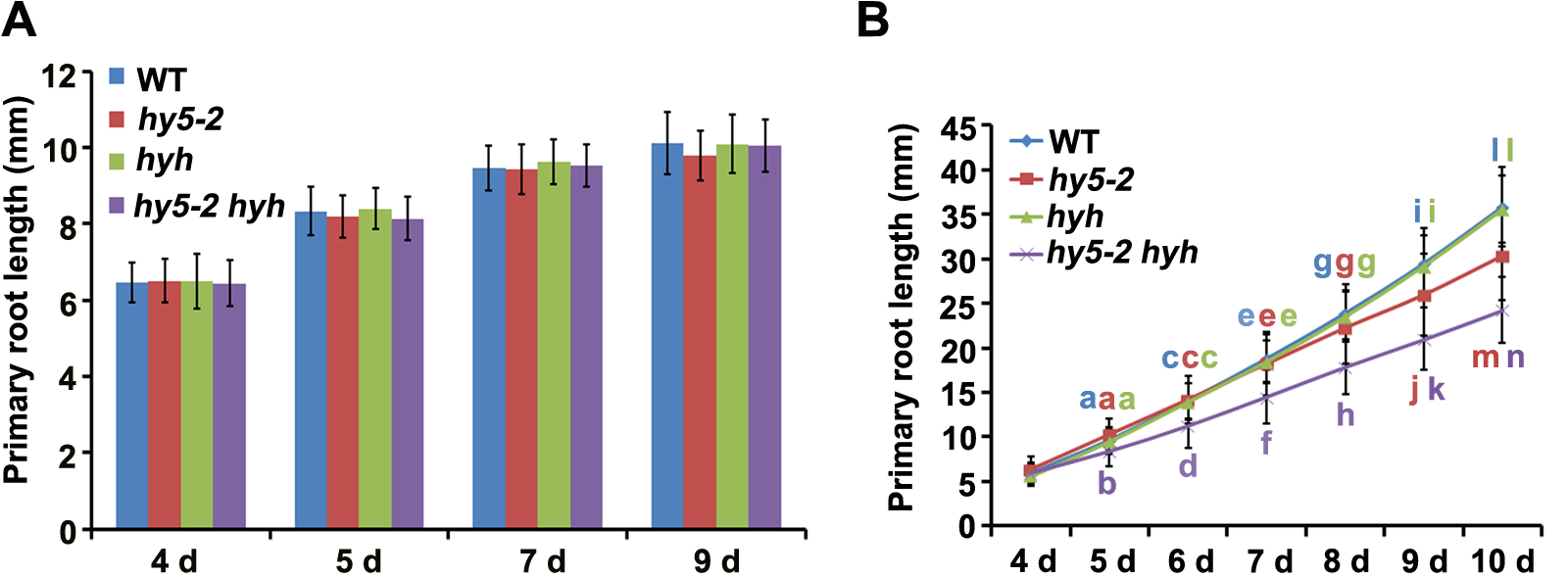

Supplement: S2 Fig — (A) Primary root length of the wild-type (WT), hy5-2, hyh, and hy5-2 hyh lines grown in darkness (n = 20). Error bars represent SD. (B) Primary root length of the WT, hy5-2, hyh, and hy5-2 hyh lines in the light (n>20). Error bars represent SD. Bars with different letters are significantly different at p<0.05 (t test). (TIF) [file pone.0180449.s002.tif]

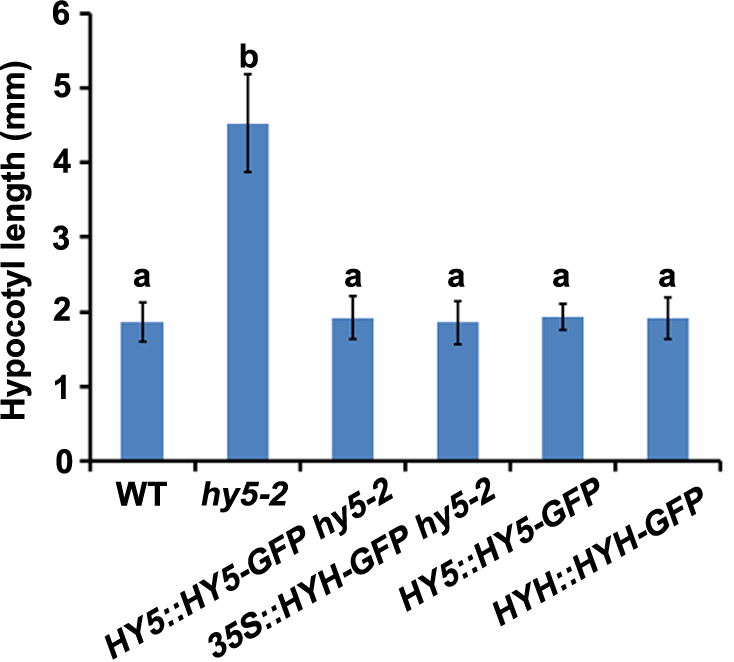

Supplement: S3 Fig — The hypocotyl length of 6-d-old light-grown wild type (WT), hy5-2 mutant, and transgenic seedlings in the WT or hy5-2 mutant background. Error bars represent SD (n>40). Bars with different letters are significantly different at p<0.05 (t test). (TIF) [file pone.0180449.s003.tif]

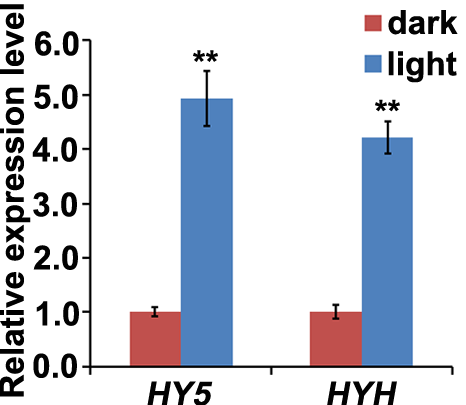

Supplement: S4 Fig — qRT-PCR quantification of HY5 and HYH transcripts. Error bars represent SD from three independent experiments. HY5 and HYH transcripts were normalized to the EF1α gene. ** P< 0.01, t test. (TIF) [file pone.0180449.s004.tif]

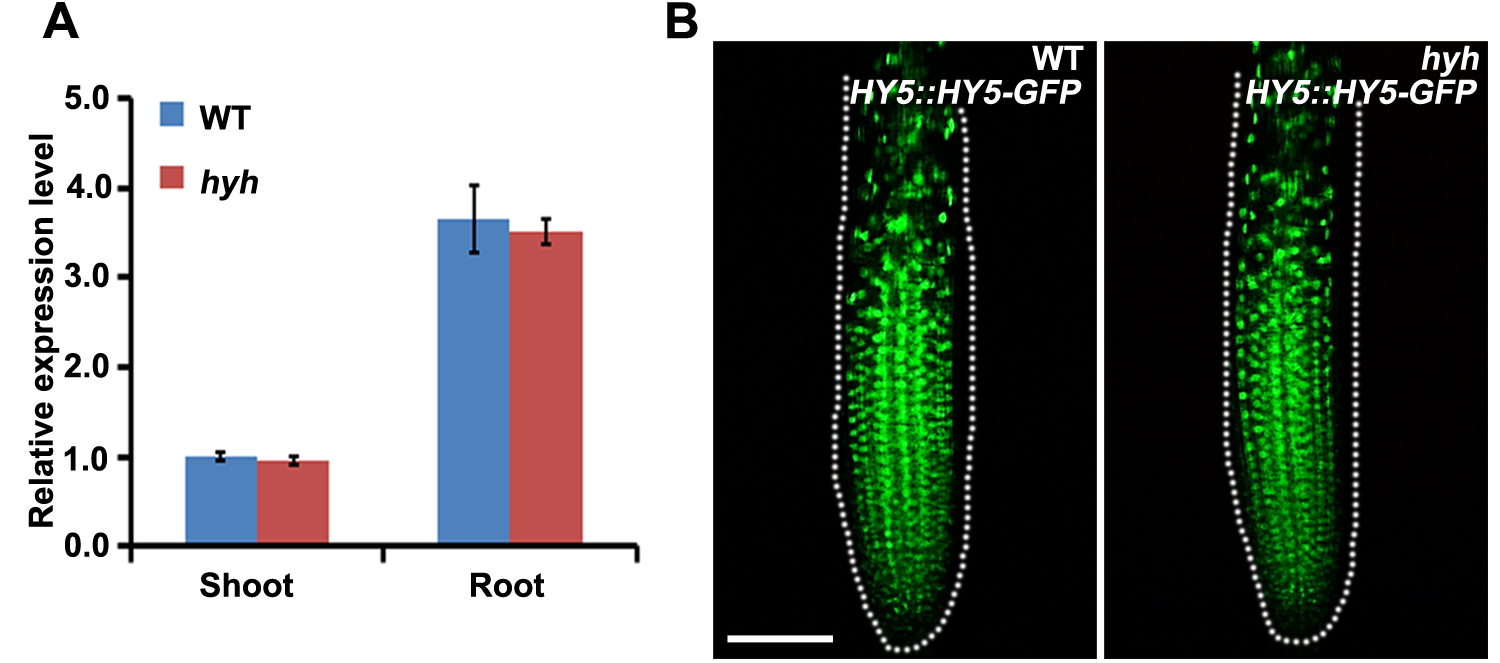

Supplement: S5 Fig — (A) qRT-PCR quantification of HY5 transcripts in the shoots and roots of wild-type and hyh plants. Error bars represent SD from three independent experiments. HY5 transcripts were normalized to the EF1α gene. (B) Expression pattern of the HY5 protein fusion line in roots of wild-type and hyh plants. The dotted line indicates the root outline. Scale bar: 50 μm. (TIF) [file pone.0180449.s005.tif]

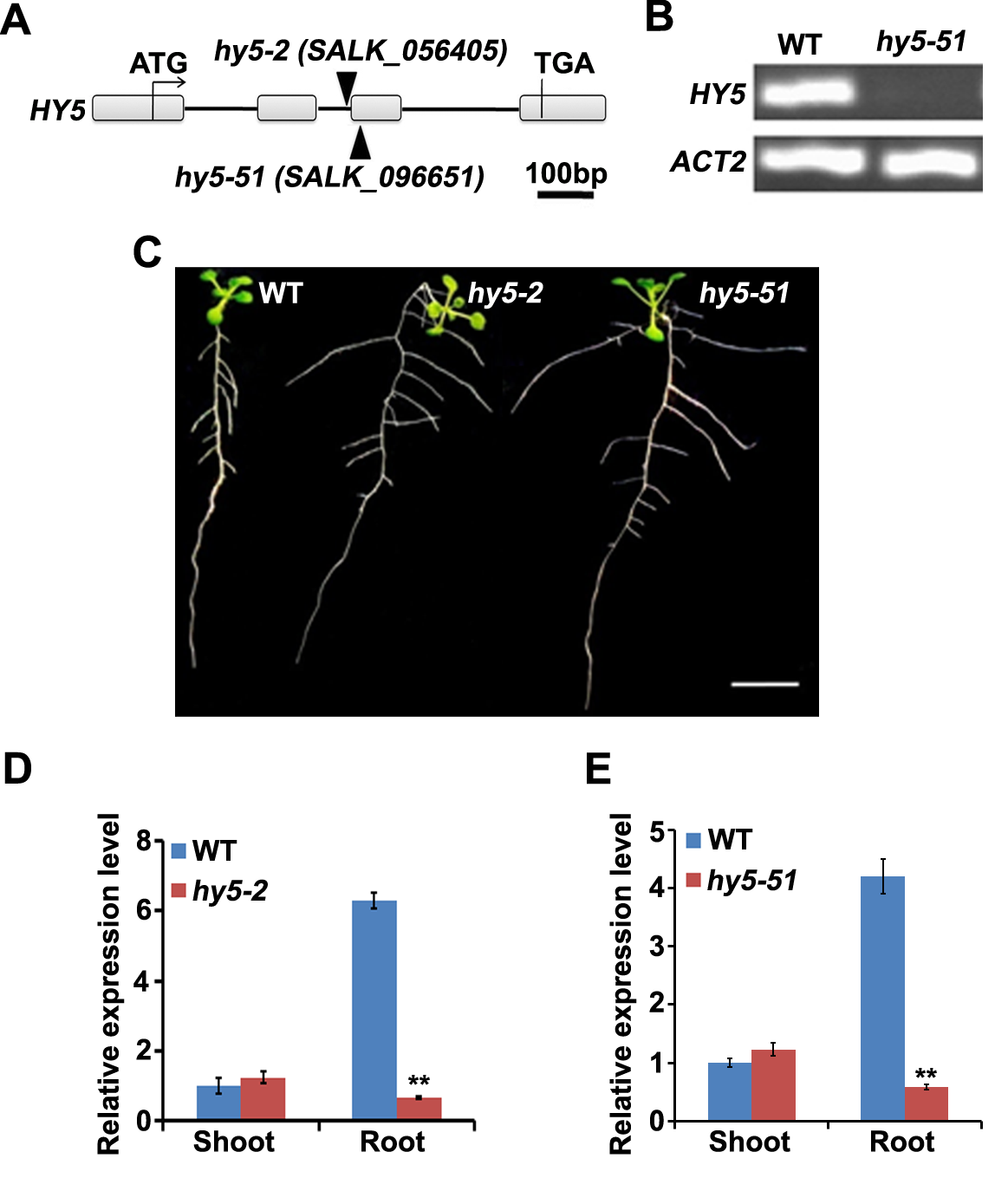

Supplement: S6 Fig — (A) Schematic diagram showing the genomic structure of HY5. Gray boxes represent exons and horizontal lines represent introns. Arrowheads indicate the location of T-DNA insertion sites in the HY5 gene. ATG, start codon; TGA, stop codon. (B) Reverse transcriptase polymerase chain reaction (RT-PCR) analysis of HY5 expression in wild-type (WT) plants and the hy5-51 mutant. ACT2 serves as reference. (C) Lateral root phenotypes of 12-d-old light-grown WT, hy5-2, and hy5-51 seedlings. Scale bar: 1 cm. (D,E) qRT-PCR quantification of HYH transcripts in the shoots and roots of the WT and hy5 mutant. Error bars represent SD from three independent experiments. HYH transcripts were normalized to the EF1α gene. ** P< 0.01, t test. (TIF) [file pone.0180449.s006.tif]
