# Supplementary material for: Dissection of HY5/HYH expression in Arabidopsis reveals a root-autonomous HY5-mediated photomorphogenic pathway
Source: PLoS One. 2017 Jul 6;12(7):e0180449. doi: 10.1371/journal.pone.0180449 (PMC5500333; doi:10.1371/journal.pone.0180449)
Supplement: S1 Table — (DOCX) [file pone.0180449.s007.docx]

**Table S1. Primers used in the study.**

| Primer name | Sequence | Purpose |
| --- | --- | --- |
| **RT-PCR & qRT-PCR** | | |
| HY5 RT-PF2 | GAGGAGAAGCTGTCGGAAAA | HY5 |
| HY5 RT-PR2 | CTCTGTTTTCCAACTCGCTCA | [51] |
| HYHRTPF | CCCACAAGAAGCACAAAACTGAGGAAA |  |
| HYHRTPR | CTTCCACGGCGGCGTTTAGCTGTAGAGA |  |
| EF1a-qPF | TGAGCACGCTCTTCTTGCTTTCA | Reference gene |
| EF1a-qPR | GGTGGTGGCATCCATCTTGTTACA | [52] |
| ACT2-qF | GGCTCCTCTTAACCCAAAGGC |  |
| ACT2-qR | CACACCATCACCAGAATCCAGC |  |
| **Cloning** | | |
| 5pF | CACGGTACCTCTAATGTTAACGTTGAGATG | HY5 protein fusion |
| 5pR | GGGAAGGCTTGCATCAGCATTAG |  |
| HpF | CACGGTACCTAATGAGACCTTGAAGCTGCAG | HYH protein fusion |
| HpR | GGGGTGATTGTCATCAGTTTTAGG |  |
| HY5pF | CACGGTACCTCTAATGTTAACGTTGAGATG | *HY5* promoter fusion |
| HY5pR | CACCTCGAGTTTTCTTACTCTTTGAAGATC |  |
| HYHpF | CACGGTACCTAATGAGACCTTGAAGCTGCAG | *HYH* promoter fusion |
| HYHpR | CACCTCGAGCAATATCTTTTTGTCTTTTGAG |  |
| **Genotyping** | | |
| hy5-2MLP | TTCACTCTCGATATCCGTTCG | SALK_056405 |
| hy5-MRP | ATGCGAGTGAATGACCATTTC |  |
| hy5-51MLP | ATTCCTTCCCAAAATGTCTCG | SALK_096651 |
| atLBb1.3 | ATTTTGCCGATTTCGGAAC |  |
| hyh-wc LP | ACTCGCATAAGAACATGTGGG | WISCDSLOX253D10 |
| hyh-wc RP | ACCCACACGCTCTGTGAATAC |  |
| p745 | AACGTCCGCAATGTGTTATTAAGTTGTC |  |
| **Y1H** |  |  |
| HY5CDS-PF-EcoR1 | TATGAATTCATGCAGGAACAAGCGACTAG | HY5-AD |
| HY5CDS-PR-Xhol1 | ACACTCGAGTCAAAGGCTTGCATCAGCATTAGAAC |  |
| HYH-cis-1-PF | TATGAATTCAAACTTTAAAATTATGATG | frag 1 |
| HYH-cis-PR | ACACTCGAGcaatatctttttgtcttttg |  |
| HYH-cis-5-PF | TATGAATTCTTGCATACAGCTTCAAATA | frag 2 |
| HYH-cis-5-PR | ACACTCGAGTTCATCATAATTTTAAAGT |  |
| HYH-cis-4-PF | TATGAATTCAAATATGATCACCATGGTTTG | frag 3 |
| HYH-cis-4-PR | ACACTCGAGCTTTTATACGAAGTACTATCC |  |
| HYH-cis-7-PF | TATCAATTGGTAGACTTGAAGAGTTTTG | frag 4 |
| HYH-cis-7-PR | ACACTCGAGGTTTAATTGATTATAGACT |  |
| **EMSA** |  |  |
| ecis3-1F | AGTTCTAACCATGGTAAATTTAAACGTTTTGTTGAAGTAATATAGAGAGG | 3’-FAM |
| ecis3-2F | CATGATATGTACTCCTAAATTAACGTGGCATGTTCATGCATAGACAAAGA | 3’-FAM |
| ecis3-3F | CAAAGATGCAATCATATCTCCACGTTTGCATACAGCTTCAAATAATAAAA | 3’-FAM |
| ccis3-1F | GTTCTAACCATGGTAAATTTAAACGTTTTGTTGAAGTAATATAGAGAGG |  |
| ccis3-2F | CATGATATGTACTCCTAAATTAACGTGGCATGTTCATGCATAGACAAAGA |  |
| ccis3-3F | CAAAGATGCAATCATATCTCCACGTTTGCATACAGCTTCAAATAATAAAA |  |
| ccis3-1R | CCTCTCTATATTACTTCAACAAAACGTTTAAATTTACCATGGTTAGAACT |  |
| ccis3-2R | TCTTTGTCTATGCATGAACATGCCACGTTAATTTAGGAGTACATATCATG |  |
| ccis3-3R | TTTTATTATTTGAAGCTGTATGCAAACGTGGAGATATGATTGCATCTTTG |  |
